# Reply to the Letter to the Editor: Quantitative accuracy of virtual monoenergetic images from multi-energy CT

**DOI:** 10.1007/s00330-023-10286-y

**Published:** 2023-11-07

**Authors:** Joel Greffier, Yoad Yagil, Klaus Erhard, Philippe C. Douek, Salim A. Si-Mohamed

**Affiliations:** 1 IMAGINE UR UM 103, Montpellier University, Department of Medical Imaging, Nîmes University Hospital, Nîmes, France; 2 Philips Healthcare, Haifa, Israel; 3 Philips Healthcare, Hamburg, Germany; 4 University Lyon, INSA-Lyon, University Claude Bernard Lyon 1, UJM-Saint Etienne, CNRS, Inserm, CREATIS UMR 5220, U1206, F‐69621, 7 Avenue Jean Capelle O, 69100 Villeurbanne, France; 5 Department of Cardiothoracic Radiology, CHU Cardiologique Louis Pradel, 59 Boulevard Pinel, 69500 Bron, France

Dear Professor Menu,

We agree with the content of the letter referring to our article entitled “Virtual monochromatic images for coronary artery imaging with a spectral photon-counting CT (SPCCT) in comparison to dual-layer CT systems: a phantom and a preliminary human study” published in *European Radiology.*

Iodine CT numbers in virtual monoenergetic images (VMIs) should not depend on the CT system. Theoretically, different CT systems, different technologies, different patient attenuation, and different acquisition protocols should all display similar CT numbers at any given keV [1]. However, we note that discrepancies between CT systems can be expected as the two-base model is only an approximation. One cannot precisely fit the energy-dependent attenuation of different materials by a linear combination of any two-base model, in general, and particularly at low VMIs. This has been shown in studies assessing dual-energy CT platforms from different vendors [2, 3]. On the contrary, iodine CT numbers in conventional images, derived from multi-energy data, do depend on the aforementioned factors. Therefore, we agree that our explanation in the discussion section is only correct for conventional images but not for VMIs.

In our study, we wish to highlight the fundamental purpose of Figure 3a. This figure was meant to provide the difference in contrast, between an insert and the phantom’s material background as found in the Mercury v4.0 phantom (Gammex). Therefore, in our study, we chose the iodine insert at 10 mg/ml so the contrast reached similar attenuation as found in the coronary lumen, i.e., ~ 350 HU at 120 kVp [4]. Now, this contrast takes into account the detectability index calculation using a non-prewhitening observer model with an eye filter, representing the figure of merit of our study [5, 6]. Hence, while Fig. 3a was not meant to provide an accurate analysis of CT numbers in VMIs, we agree that it suggests variability between SPCCT and EID-DLCT CT numbers.

Therefore, as suggested, we performed a quantitative comparison of CT numbers measured on VMIs for energy levels ranging from 40 to 90 keV. To do this, acquisitions were performed on the Multi-energy CT phantom (Gammex, Sun Nuclear Corporation, WI) for the same acquisition and reconstruction parameters as those used in our study (Table 1) [7]. Five acquisitions were performed on a clinical SPCCT prototype (Philips) and an EID-DLCT system (CT7500, Philips). For each acquisition and energy level, a region of interest of 2 cm in diameter was placed inside the iodine insert at 10 mg/ml. The mean pixel attenuation was computed for 6 energy levels on the VMIs, as performed in our study [7]. The measured CT number (${HU}_{measured}$) was then compared with its respective theoretical value (${HU}_{theoretical}$) for each keV. The relative error [%] between theoretical and measured numbers was calculated between the attenuation coefficient µ, using the following formula:

**Table 1:** CT scan parameters for quantitative VMI comparison

	EID-DLCT (CT7500)	SPCCT
Tube voltage (kVp)	120	120
Tube current time-product (mAs)	255	255
Rotation time (s)	0.27	0.33
Pitch	0.2	0.318
CTDIvol (mGy)	25	25
Collimation (rows x mm)	128 × 0.625	64 × 0.2750
Reconstruction kernel	CB (cardiac standard)	Detailed 2/HRB
Slice thickness (mm)	0.67	0.25
Field of view (mm)	220	220
Matrix size	512	1024
VMI range (keV)	40 to 90 (10 keV steps)	40 to 90 (10 keV steps)

*EID-DLCT*, energy integrating detector dual-layer computed tomography; *SPCCT*, spectral photon-counting detector computed tomography; *CTDIvol*, volume CT dose index; *VMI*, virtual monoenergetic image

$$Relative\; error\, \left(\%\right)=100\times \frac{{\mu }_{measured}-{\mu }_{theoretical}}{{\mu }_{theoretical}}=100\times \frac{\left(1000+{HU}_{measured}\right)-\left(1000+{HU}_{theoretical}\right)}{1000+{HU}_{theoretical}}$$

As shown in Table 2**,** the mean relative errors of SPCCT and EID-DLCT were in the same range as the reference values (mean error < 1%). Nevertheless, we noted a larger standard deviation for SPCCT which illustrated imperfection according to the energy levels, particularly at the lowest and highest energy levels. This deviation may be due to the status of spectral processing in the current prototype system which is still under development.

**Table 2:** Mean relative errors obtained for energy levels from 40 to 90 keV between measured CT number of iodine insert at 10 mg/ml and its respective theoretical value for an energy integrating detector dual-layer computed tomography (EID-DLCT) and a clinical prototype spectral photon-counting detector computed tomography (SPCCT)

	EID-DLCT	SPCCT	SPCCT
Energy levels	Filter XCB (cardiac standard)	Filter HRB	Filter Detailed 2
40 keV	1.1% ± 0.2%	− 2.2% ± 0.5%	− 2.1% ± 0.4%
50 keV	0.3% ± 0.1%	− 1.7% ± 0.2%	− 1.7% ± 0.2%
60 keV	0.0% ± 0.1%	− 0.6% ± 0.1%	− 0.6% ± 0.1%
70 keV	− 0.1% ± 0.1%	0.5% ± 0.2%	0.5% ± 0.2%
80 keV	− 0.2% ± 0.1%	1.3% ± 0.3%	1.4% ± 0.2%
90 keV	− 0.2% ± 0.1%	2.0% ± 0.4%	2.0% ± 0.2%
**Overall**	**0.1% ± 0.5%**	** − 0.1% ± 1.6%**	** − 0.1% ± 1.6%**

Overall energy levels

Finally, and more importantly, we wish to point out that the main conclusion of our study is still valid for 2 reasons. First, taking into account the contrast calculated in the manuscript, the detectability index of SPCCT images remains far better than the reference EID-DLCT images. For example, while the contrast at 40 keV was 14 ± 1% higher than in the reference images, the detectability was 111% ± 9% higher using a similar reconstruction kernel (HRB vs CB) and slice thickness (0.67 mm). Second, taking into account the theoretical CT numbers of the iodine insert at 10 mg/ml into the calculation of the detectability index, the values of SPCCT were 91% ± 15% far greater than EID-DLCT (Fig. 1). Altogether, these results are mainly explained by the technical benefits of SPCCT providing a higher spatial resolution, a shift towards higher spatial frequency of the noise, and a lower noise magnitude.

**Fig. 1:**
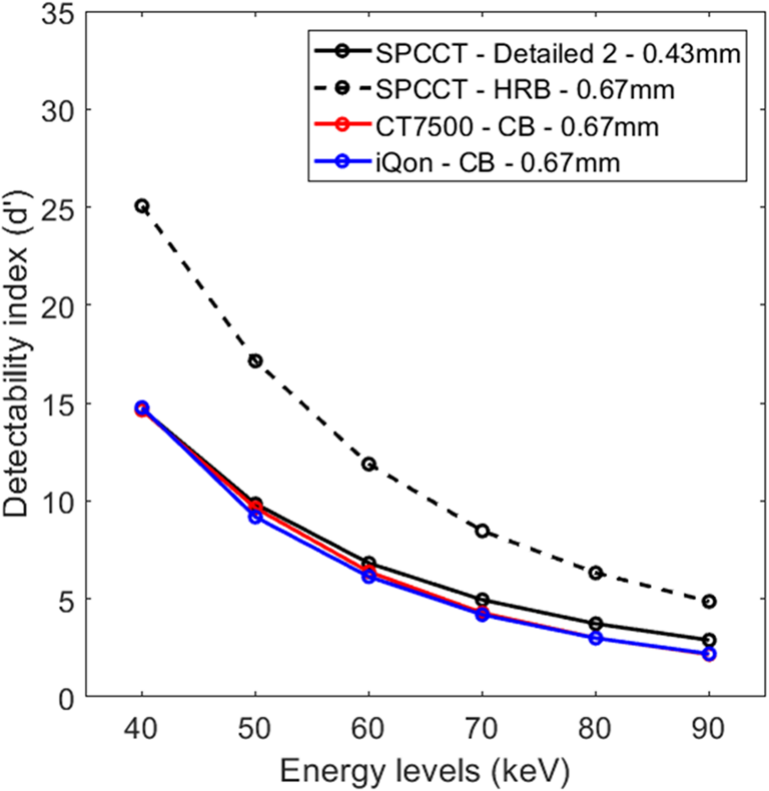
Detectability index (d’) values of the simulated lesion according to the energy levels from 40 to 90 keV on the two energy-integrating detector dual-layer computed tomography systems (CT7500 and iQon) and a clinical prototype spectral photon-counting detector computed tomography (SPCCT). *d’ values were computed using the theoretical HU values of the iodine insert of 10 mg/mL for each energy level: 843 HU for 40 keV, 555 HU for 50 keV, 375 HU for 60 keV, 263 HU for 70 keV, 193 HU for 80 keV, 146 HU for 90 keV*

In conclusion, the letter referring to our article reflects the fact that the current clinical SPCCT prototype is still under development with routine upgrades and that additional work is needed to achieve the full potential that SPCCT technology may bring in future in the radiology field [4, 8–10].

## Declarations

### Guarantor

The scientific guarantor of this publication is Pr. Philippe Douek.

### Statistics and biometry

No complex statistical methods were necessary for this manuscript.

### Study subjects or cohorts overlap

N/A.

### Methodology

• Experimental for phantom study.

• Performed at one institution.

## References

[1] Alvarez RE, Macovski A (1976). Energy-selective reconstructions in X-ray computerized tomography. Phys Med Biol.

[2] Sellerer T, Noël PB, Patino M (2018). Dual-energy CT: a phantom comparison of different platforms for abdominal imaging. Eur Radiol.

[3] Jacobsen MC, Cressman ENK, Tamm EP (2019). Dual-energy CT: lower limits of iodine detection and quantification. Radiology.

[4] Si-Mohamed S, Boccalini S, Lacombe H (2022). Coronary CT angiography with photon-counting CT: first-in-human results. Radiology.

[5] Samei E, Bakalyar D, Boedeker KL (2019). Performance evaluation of computed tomography systems: summary of AAPM Task Group 233. Med Phys.

[6] Greffier J, Barboteau Y, Gardavaud F (2022). iQMetrix-CT: New software for task-based image quality assessment of CT images. Diagn Interv Imaging.

[7] Greffier J, Si-Mohamed SA, Lacombe H et al (2023) Virtual monochromatic images for coronary artery imaging with a spectral photoncounting CT in comparison to dual-layer CT systems: a phantom and preliminary Human study. Eur Radiol 33:5476–5488. 10.1007/s00330-023-09529-9 10.1007/s00330-023-09529-9 PMC10326132 36920517

[8] Si-Mohamed SA, Greffier J, Miailhes J (2021). Comparison of image quality between spectral photon-counting CT and dual-layer CT for the evaluation of lung nodules: a phantom study. Eur Radiol.

[9] Si-Mohamed SA, Sigovan M, Hsu JC (2021). In vivo molecular K-edge imaging of atherosclerotic plaque using photon-counting CT. Radiology.

[10] Greffier J, Villani N, Defez D (2022). Spectral CT imaging: Technical principles of dual-energy CT and multi-energy photon-counting CT. Diagn Interv Imaging.

